# Myocardial Performance Index in Neurocardiogenic Syncope Patients

**DOI:** 10.14740/cr367w

**Published:** 2014-12-04

**Authors:** Fatma Yilmaz Coskun, Murat Sucu, Okkes Uku, Murat Yuce, Orhan Ozer, Suleyman Ercan, Vedat Davutoglu

**Affiliations:** a Cardiology Department, Gaziantep University, Gaziantep, Turkey; b Cardiology Department, Elazig Research and Training Hospital, Elazig, Turkey

**Keywords:** Myocardial performance index, Neurocardiogenic syncope, Echocardiography

## Abstract

**Background:**

Many syncopes resulting from neural reflexes in various conditions are called neurocardiogenic syncope (NCS). We aimed to investigate the presence of left ventricular (LV) myocardial performance index (MPI) in patients with NCS, which was diagnosed with head-up tilt table test (HUTT), and the accurateness of the test in order to use it as a method in patients with NCS. Assuming the MPI as a potential cause of syncope, we assessed the Tei index with non-invasive tissue Doppler echocardiography method.

**Methods:**

Consecutive outpatients with a history of recurrent unexplained syncope underwent HUTT. Twenty-nine HUTT (+) patients (24 female and five male, mean age: 30 ± 15 years) as the study group and HUTT (-) 23 healthy patients (six female and 17 male, mean age: 34 ± 16 years) as the control group were included into the study. Conventional and tissue Doppler echocardiography was performed to both groups. The MPI was determined by using PW Doppler. Measurements of Doppler time intervals, according to Tei index ((isovolumic contraction time + isovolumic relaxation time)/ejection time) is calculated as (a - b/b), where “a” is the interval between cessation and onset of the mitral inflow, and “b” is the ejection time (ET) at the LV outflow.

**Results:**

When comparing the groups in terms of MPI and ET, there was significant difference between groups. Patients with NCS had significantly longer ET and lower MPI value than control group (284 ± 24 ms vs. 260 ± 24 ms, P < 0.001, respectively and 0.44 ± 0.7 vs. 0.52 ± 0.8, P < 0.001, respectively). There was no significant difference in ejection fraction between groups.

**Conclusion:**

In the present study, LV MPI value decreases in patients with NCS.

## Introduction

Syncope is defined as a transient, sudden loss of muscle tone with loss of consciousness, the onset of which is relatively rapid, and where the subsequent recovery is usually prompt and complete. The underlying mechanism is a temporary global cerebral hypoperfusion. Neurocardiogenic syncope (NCS) is a disorder of autonomic regulation of cardiovascular reflexes which results vasodilation and/or bradycardia that causes loss of consciousness and postural tone. NCS refers to a neurally mediated response that gives rise to vasodilatation and/or bradycardia [1]. NCS in the diagnosis of head-up tilt table test (HUTT) is the most reliable method. Myocardial performance index (MPI), also known as Tei index, has been studied as a measure of global myocardial performance, being theoretically linked to both the systolic and the diastolic component of the cardiac cycle [2]. Many studies have in fact demonstrated the diagnostic and prognostic reliability of MPI in many different clinical conditions.

## Patients and Methods

### Study group

Fifty-two patients who were admitted to the cardiology outpatient clinics with complaints of syncope or presyncope between January 2011 and November 2011, aged between 18 and 70, and who underwent HUTT for diagnostic purposes were included in the study. The patients were divided into two groups according to the HUTT (+) or HUTT (-) test results. The exclusion criteria were known coronary artery disease, arterial hypertension, left ventricular (LV) wall anomalies, ejection fraction (EF) below 50%, primary cardiomyopathy, valvular heart disease, non-sinus rhythm on the electrocardiography (ECG), bundle branch block and atrioventricular block, thyroid dysfunction, renal failure, and a history of drug abuse. Medical examination included anthropometric measurements, body height, body mass, and body mass index (BMI) calculated according to the following formula: BMI = body mass (kg)/(body height)^2^. Body surface area (BSA) is calculated by the following formula: BSA = (W^0.425^ × H^0.725^) × 0.007184 [3].

### HUTT method

All patients fasted for at least 4 h for the HUTT. A venous access was provided through the antecubital vein in all patients. Continuous ECG monitoring (DII derivation) and automatical arterial blood pressure measurements were performed in all patients. After obtaining the baseline heart rate and blood pressure, the standard HUTT was performed in all patients [4]. Passive phase was applied at the tilt table, tilted to 70°, for at least 20 min. In patients with a negative result after the passive tilt, 0.4 mg of nitroglycerin was given sublinguinally and the test was continued until the syncope occurred or until the protocol terminated (25 min after the medication was given, a total of 45 min). An arterial systolic blood pressure of 80 mm Hg or lower and/or bradycardia or asystole development together with syncope or presyncope was considered as a positive response [5]. All patients who agreed to participate in the study were informed about the study protocol, and the written and the verbal consent were obtained. The medical ethical review committees of the participating hospitals approved the study. Informed consent was obtained from all subjects.

### Statistical analysis

SPSS 15.0 statistical program was used for the evaluation and testing of the variables (IBM Corp., Armonk, NY, USA). For the comparison of the patients who developed vasovagal syncope as a result of HUTT and the group with a negative test result, the Student’s *t*-test was used for the parameters with a normal distribution. The Mann-Whitney U test was used for the parameters without a normal distribution. Numerical variables were given as mean ± standard deviation and categorical variables were expressed as percent.

### Conventional echocardiographic examination

After the HUTT test, in all patients, a transthoracic echocardiography was performed on the same day by a cardiologist who did not know the patient’s clinical status. The echocardiographic examinations were performed in the left lateral and supine positions, using the Vivid 7 (GE Vingmed Ultrasound, Horten, Norway) 2 - 4 MHz transducer and under the continuous monitoring with the DII derivation. The measurements and the evaluations were done according to the American Society of Echocardiography appropriate use criteria [6]. The LV systolic and diastolic diameters, interventricular septum, and posterior wall diastolic diameter were measured using the M-mode method with the parasternal long axis imaging. For the imaging of the anteroposterior diameter of the left atrium in the parasternal long axis view, the distance between the posterior wall of the aorta and the posterior wall of the left atrium at the end of systole was measured. In the apical four-chamber view, the sample volume was placed between the mitral leaflet tips, and the internal mitral flow velocity-time curve was obtained with the pulsed-wave (PW) Doppler method. Using this curve, the mitral peak velocities of the E and A waves were measured. All these measurements were performed in consecutive cardiac cycles and the average of three measurements was calculated.

### Tissue Doppler imaging

Tissue Doppler echocardiography was performed using 3.5 - 4.0 MHz transducers, adjusting the spectral pulsed Doppler signal filter to a Nyquist limit of 15 - 20 cm/s, with a minimal optimal gain. The velocity of the monitor was set between 50 mm/s and 100 mm/s with optimal spectral images of the myocardial velocities. In the apical four-chamber view, the tissue Doppler images were obtained by placing the PW Doppler cursor in the left lateral mitral annulus, septal mitral annulus, and right ventricular tricuspid annulus. PW Doppler cursor was placed with a perpendicular Doppler angle. The MPI was determined by using PW Doppler. Measurement of Doppler time intervals, according to Tei index ((IVCT + IVRT)/ET) is calculated as (a - b/b), where “a” is the interval between cessation and onset of the mitral inflow, “b” is the ejection time (ET) at the LV outflow, IVCT is the isovolumic contraction time and IVRT is the isovolumic relaxation time [2].

## Results

The distribution of the 29 patients who developed vasovagal syncope in the HUTT (+) and 23 subjects in control group with a negative HUTT (-), according to age and gender is given in Table 1. The mean age was 30.6 ± 15.9 years in the HUTT (+), and 34.7 ± 16.3 years in the HUTT (-) group (P > 0.05). There were 24 female and five male patients in the HUTT (+) group, and 17 men and six women in the control group (P = 0.0001). The number of women in the vasovagal syncope group was significantly higher compared to the control group (P = 0.0001). The patients in the HUTT (+) group were shorter than the patients in the control group (P = 0.006) with a lower BSA (P < 0.05).

**Table 1:** Patient Characteristics and Baseline Echocardiographic Findings

	HUTT (+) (n = 29)	HUTT (-) (n = 23)	P value
Gender (female/male)	24/5	6/17	0.0001
Age (years, mean ± SD)	30.6 ± 15.9	34.7 ± 16.3	NS
Height (cm)	165.1 ± 7.6	171.4 ± 8	0.006
Weight (kg)	67.0 ± 13.8	73.3 ± 14.2	NS
BSA (kg/m^2^)	24.6 ± 5.6	27.9 ± 6.2	0.05
Left ventricular diameter (mm)	41.8 ± 3.8	42.7 ± 3.3	NS
Aortic diameter (mm/m^2^)	15.88 ± 2.04	16.82 ± 1.95	NS
Left atrial diameter (mm)	29.8 ± 3.4	31.2 ± 4.4	NS
Right atrial diameter (mm)	31.7 ± 3.9	31.1 ± 7.4	NS

BSA: body surface area; HUTT (+): head-up tilt test positive patients; HUTT (-): head-up tilt table test negative patients; NS: not significant, P < 0.05.

In the patient group compared to the control group, the conventional Doppler flow and the early diastolic mitral flow (E) were significantly higher in HUTT (+) patients (Table 2). There were no statistically significant differences between the groups in terms of left atrial diameter, right atrial diameter, LV diameter, aortic diameter, age, and weight. There was also no statistically significant difference between the two groups in terms of the IVRT (P > 0.05), whereas the difference was statistically significant with regard to MPI (P = 0.01), ICRT (P = 0.02) and ET (P = 0.001) (Table 2).

**Table 2:** Tissue Doppler and Conventional Echocardiographic Findings in Patients

	HUTT (+) (n = 29)	HUTT (-) (n = 23)	P value
E/Em	5.83 ± 2.24	5.96 ± 2.36	NS
Septal (cm/s)			
Sm	10.6 ± 2.4	9.3 ± 1.7	0.02
Em	16.3 ± 3.5	13.3 ± 5.5	0.05
Am	11.5 ± 3.7	10.6 ± 2.7	NS
Mitral E (m/s)	0.83 ± 0.1	0.69 ± 0.1	0.03
Mitral A (m/s)	0.64 ± 0.1	0.63 ± 0.1	NS
Mitral E/A	1.3 ± 0.3	1.1 ± 0.4	NS
IVCT (ms)	46.6 ± 9.32	54.69 ± 8.07	0.002
IVRT (ms)	77.77 ± 14.19	82.27 ± 15.78	NS
ET (ms)	284 ± 24.68	260.43 ± 24.47	0.001
MPI	0.44 ± 0.7	0.52 ± 0.8	0.001

HUTT (+): head-up tilt test positive patients; HUTT (-): head-up tilt table test negative patients; NS: not significant, P < 0.05.

In the correlation analysis, there was a negative correlation between the MPI and early diastolic mitral flow (E) (r = -540, P = 0.001) and E/Em (r = -390, P = 0.007), and a positive correlation between aortic diameter (r = 0.594, P = 0.001), BMI (r = 0.365, P = 0.009), BSA (r = 0.334, P = 0.01) and age (r = 0.475, P = 0.001). In addition, a positive correlation was found between the MPI and LV diameter (r = 0.352, P = 0.01) and left atrial diameter (r = 0.357, P = 0.01). There was a negative correlation between the ET and height (r = -0.284, P = 0.04), whereas a positive correlation was found between early diastolic mitral flow (E) (r = 0.437, P = 0.002).

## Discussion

In this study, we aimed to test whether the MPI is a pathophysiological determinant of the patients with NCS. This study has revealed for the first time that in patients with vasovagal syncope, the Tei index was significantly better in patients with NCS.

Structural heart disease does not cause loss of consciousness and posture NCS, often resulting in LV depending on the stimulation of mechanoreceptors Bezold-Jarish reflex [7, 8]. The stimulation of mechanoreceptors and venous congestion, sympathetic nervous system activation and excessive LV contraction is observed. Tilt table test in patients with a positive response to an increase in LV fractional shortening and LV volume reduction in patients with NCS shows warned of LV mechanoreceptors. The enhanced adrenergic tone increases myocardial contractile force and heart rate to compensate for the reduced stroke volume [9]. Syncope induced by head-up tilt is associated with a strong myocardial contraction and a significant reduction in the end-systolic LV size. The LV hypercontractilily and decreased LV diameters may play an important role in the pathogenesis of syncope induced by head-up tilt test [10]. The activating unmyelinated LV vagal nerve endings are known as mechanoreceptors or C fibers. The LV myocardial contraction around an empty LV cavity leads to stimulation of C fibers and therefore, vasodilatation, bradycardia and syncope. It is reasonable that similar mechanisms may also be operative during syncope induced by a head-up tilt test [11]. In abnormal response to head-up tilt test, LV volumes gradually decrease, along with a gradual increase in fractional shortening to > 50%. In the majority of patients, the maximal value of fractional shortening was achieved > 2 min before the onset of syncope. These findings demonstrate a hypercontracted state of the left ventricle, in which vigorous myocardial contraction occurs around an empty LV cavity [12]. In patients with vasovagal syncope, the reduction in end-diastolic volume index occurs more rapidly than in normal subjects. During the tilt, there is a more prominent reduction in the pulse index and the EF in the vasovagal group, which is possibly due to the further migration of the venous blood volume to the peripheral venous system, and the presence of an early vagal effect during the ventricular contraction [13]. Both the mitral flow (E wave) and septal mitral anulus Em wave on tissue Doppler are recorded during the rapid ventricular filling period at early diastole. The Em reflects the myocardial relaxation. In this study, Em was found to be more increased in the HUTT (+) group compared to the HUTT (-) group. Therefore, we concluded that a higher Em is associated with a better diastolic function. Su et al recently reported that Ea and E/Em were reported to be significantly correlated with time constant of isovolumic LV pressure decline and LV filling pressure in atrial fibrillation patients [14]. In this study, IVRT had a moderately strong correlation with Em and a moderate correlation with E/Em. The ET normalized to the cardiac cycle may be a hemodynamic marker of the decrease in LV during bradycardic presyncope in patients undergoing HUTT [15]. The duration of ET reflects both the velocity and extent of fiber shortening. In LV decompensation, the extent of fiber shortening is decreased, so a shortened ET is usually noted [16]. Whereas, in our study, we have found that, duration of ET was longer in the patients group than the control group. Lee et al reported that there were no significant differences in LV end-diastolic dimension and end-systolic dimension in each group between baseline and isoproterenol infusion during posture change. Vasovagal syncope was associated with vigorous myocardial contraction, rather than with contraction against an empty left ventricle [17]. Limited intracardiac volume reserve has been reported to play an important role in the mechanism of syncope, and the decreased left atrial volume was claimed to be an independent predictor of HUTT-induced syncope. In our study, we have found the diameter of the left atrium was much smaller in the patient group; this difference was not statistically significant [18]. Deharo et al reported that after the initial tilt, a significant increase in myocardial contractility was observed in the vasovagal patients and not in the control patients [19]. In our study, we have found similar findings.

To our knowledge, the present study was the first to investigate the Tei index in NCS patients using TDI echocardiography. In our study, we have found that the Tei index was significantly better in patients with NCS. Clinical implication should be studied further.

## References

[1] Benditt DG (1997). Neurally mediated syncopal syndromes: pathophysiological concepts and clinical evaluation. Pacing Clin Electrophysiol.

[2] Tei C, Ling LH, Hodge DO, Bailey KR, Oh JK, Rodeheffer RJ, Tajik AJ (1995). New index of combined systolic and diastolic myocardial performance: a simple and reproducible measure of cardiac function--a study in normals and dilated cardiomyopathy. J Cardiol.

[3] DuBois D, DuBois EF (1916). A formula to estimate the approximate surface area if height and weight be known. Arch Intern Medicine.

[4] Fitzpatrick AP, Theodorakis G, Vardas P, Sutton R (1991). Methodology of head-up tilt testing in patients with unexplained syncope. J Am Coll Cardiol.

[5] Brignole M, Menozzi C, Del Rosso A, Costa S, Gaggioli G, Bottoni N, Bartoli P (2000). New classification of haemodynamics of vasovagal syncope: beyond the VASIS classification. Analysis of the pre-syncopal phase of the tilt test without and with nitroglycerin challenge. Vasovagal Syncope International Study. Europace.

[6] Douglas PS, Garcia MJ, Haines DE, Lai WW, Manning WJ, Patel AR, Picard MH (2011). ACCF/ASE/AHA/ASNC/HFSA/HRS/SCAI/SCCM/SCCT/SCMR 2011 Appropriate Use Criteria for Echocardiography. A Report of the American College of Cardiology Foundation Appropriate Use Criteria Task Force, American Society of Echocardiography, American Heart Association, American Society of Nuclear Cardiology, Heart Failure Society of America, Heart Rhythm Society, Society for Cardiovascular Angiography and Interventions, Society of Critical Care Medicine, Society of Cardiovascular Computed Tomography, and Society for Cardiovascular Magnetic Resonance Endorsed by the American College of Chest Physicians. J Am Coll Cardiol.

[7] Abboud FM (1993). Neurocardiogenic syncope. N Engl J Med.

[8] Mark AL (1983). The Bezold-Jarisch reflex revisited: clinical implications of inhibitory reflexes originating in the heart. J Am Coll Cardiol.

[9] Paley HW, McDonald IG, Blumenthal J, Mailhot J (1971). The effects of posture and isoproterenol on the velocity of left ventricular contraction in man. The reciprocal relationship between left ventricular volume and myocardial wall force during ejection on mean rate of circumferential shortening. J Clin Invest.

[10] Shalev Y, Gal R, Tchou PJ, Anderson AJ, Avitall B, Akhtar M, Jazayeri MR (1991). Echocardiographic demonstration of decreased left ventricular dimensions and vigorous myocardial contraction during syncope induced by head-up tilt. J Am Coll Cardiol.

[11] Thoren P (1979). Role of cardiac vagal C-fibers in cardiovascular control. Rev Physiol Biochem Pharmacol.

[12] Oberg B, Thoren P (1972). Increased activity in left ventricular receptors during hemorrhage or occlusion of caval veins in the cat. A possible cause of the vaso-vagal reaction. Acta Physiol Scand.

[13] Yamanouchi Y, Jaalouk S, Shehadeh AA, Jaeger F, Goren H, Fouad-Tarazi FM (1996). Changes in left ventricular volume during head-up tilt in patients with vasovagal syncope: an echocardiographic study. Am Heart J.

[14] Su HM, Lin TH, Hsu PC, Chu CY, Lee WH, Lee CS, Lai WT (2011). Myocardial performance index derived from preejection period: a novel and feasible parameter in evaluation of cardiac performance in patients with permanent atrial fibrillation. Echocardiography.

[15] Fuca G, Dinelli M, Gianfranchi L, Bressan S, Corbucci G, Alboni P (2011). Assessment of systolic ejection time as a hemodynamic marker of incipient bradycardic vasovagal syncope. A pilot study. Pacing Clin Electrophysiol.

[16] Garrard CL, Weissler AM, Dodge HT (1970). The relationship of alterations in systolic time intervals to ejection fraction in patients with cardiac disease. Circulation.

[17] Lee TM, Chen MF, Su SF, Chao CL, Liau CS, Lee YT (1996). Excessive myocardial contraction in vasovagal syncope demonstrated by echocardiography during head-up tilt test. Clin Cardiol.

[18] Moon J, Shim J, Park JH, Hwang HJ, Joung B, Ha JW, Lee MH (2013). Small left atrial volume is an independent predictor for fainting during head-up tilt test: the impact of intracardiac volume reserve in vasovagal syncope. Int J Cardiol.

[19] Deharo JC, Peyre JP, Chalvidan T, Thirion X, Valli M, Ritter P, Djiane P (2000). Continuous monitoring of an endocardial index of myocardial contractility during head-up tilt test. Am Heart J.

